# Pain and the Triple Network Model

**DOI:** 10.3389/fneur.2022.757241

**Published:** 2022-03-07

**Authors:** Dirk De Ridder, Sven Vanneste, Mark Smith, Divya Adhia

**Affiliations:** ^1^Section of Neurosurgery, Department of Surgical Sciences, Dunedin School of Medicine, University of Otago, Dunedin, New Zealand; ^2^School of Psychology, Global Brain Health Institute, Institute of Neuroscience, Trinity College Dublin, Dublin, Ireland; ^3^Neurofeedbackservices of New York, New York, NY, United States

**Keywords:** pain, acute, chronic, cognitive, emotional, autonomic, anterior cingulate cortex, triple network

## Abstract

Acute pain is a physiological response that causes an unpleasant sensory and emotional experience in the presence of actual or potential tissue injury. Anatomically and symptomatically, chronic pathological pain can be divided into three distinct but interconnected pathways, a lateral “painfulness” pathway, a medial “suffering” pathway and a descending pain inhibitory circuit. Pain (fullness) can exist without suffering and suffering can exist without pain (fullness). The triple network model is offering a generic unifying framework that may be used to understand a variety of neuropsychiatric illnesses. It claims that brain disorders are caused by aberrant interactions within and between three cardinal brain networks: the self-representational default mode network, the behavioral relevance encoding salience network and the goal oriented central executive network. A painful stimulus usually leads to a negative cognitive, emotional, and autonomic response, phenomenologically expressed as pain related suffering, processed by the medial pathway. This anatomically overlaps with the salience network, which encodes behavioral relevance of the painful stimuli and the central sympathetic control network. When pain lasts longer than the healing time and becomes chronic, the pain- associated somatosensory cortex activity may become functionally connected to the self-representational default mode network, i.e., it becomes an intrinsic part of the self-percept. This is most likely an evolutionary adaptation to save energy, by separating pain from sympathetic energy-consuming action. By interacting with the frontoparietal central executive network, this can eventually lead to functional impairment. In conclusion, the three well-known pain pathways can be combined into the triple network model explaining the whole range of pain related co-morbidities. This paves the path for the creation of new customized and personalized treatment methods.

## Introduction

The International Association for the Study of Pain (IASP) defines pain as “an unpleasant sensory and emotional experience associated with, or resembling that associated with, actual or potential tissue damage,” or described in terms of such damage (1). Chronic pain is currently simply defined, based on temporal measures, as “pain that persists for longer than 3 months” (2–4), which extends beyond the period of healing of an acute injury. Chronic pain is thus considered an independent condition, lacking the acute warning function of physiological nociception (3).

Along with the pain itself, about 1/3 of the individuals with chronic pain present with other symptoms such as irritability, depression, anxiety, and sleep problems (5–7), as well as cognitive dysfunction, including problems of attention, learning, memory, and decision making (8). These symptoms drive most of the morbidity, leading to increased physical and functional disability and poor quality of life (9). Chronic pain is thus a significant and growing health challenge, contributing to the highest disability associated burden worldwide and accounting for significant health and non-health related costs (10). Many of the currently available pain therapies demonstrate either small treatment effect or cause uncomfortable to deleterious side effects (11, 12). Thus, new, innovative, evidenced-based, specific and safer therapies are highly needed for management of chronic pain. To develop more efficacious pain treatments a better understanding of the pathophysiological mechanisms that generate and maintain chronic pain is required.

### A Brief History of the Anatomy of Pain

**Figure 1:**
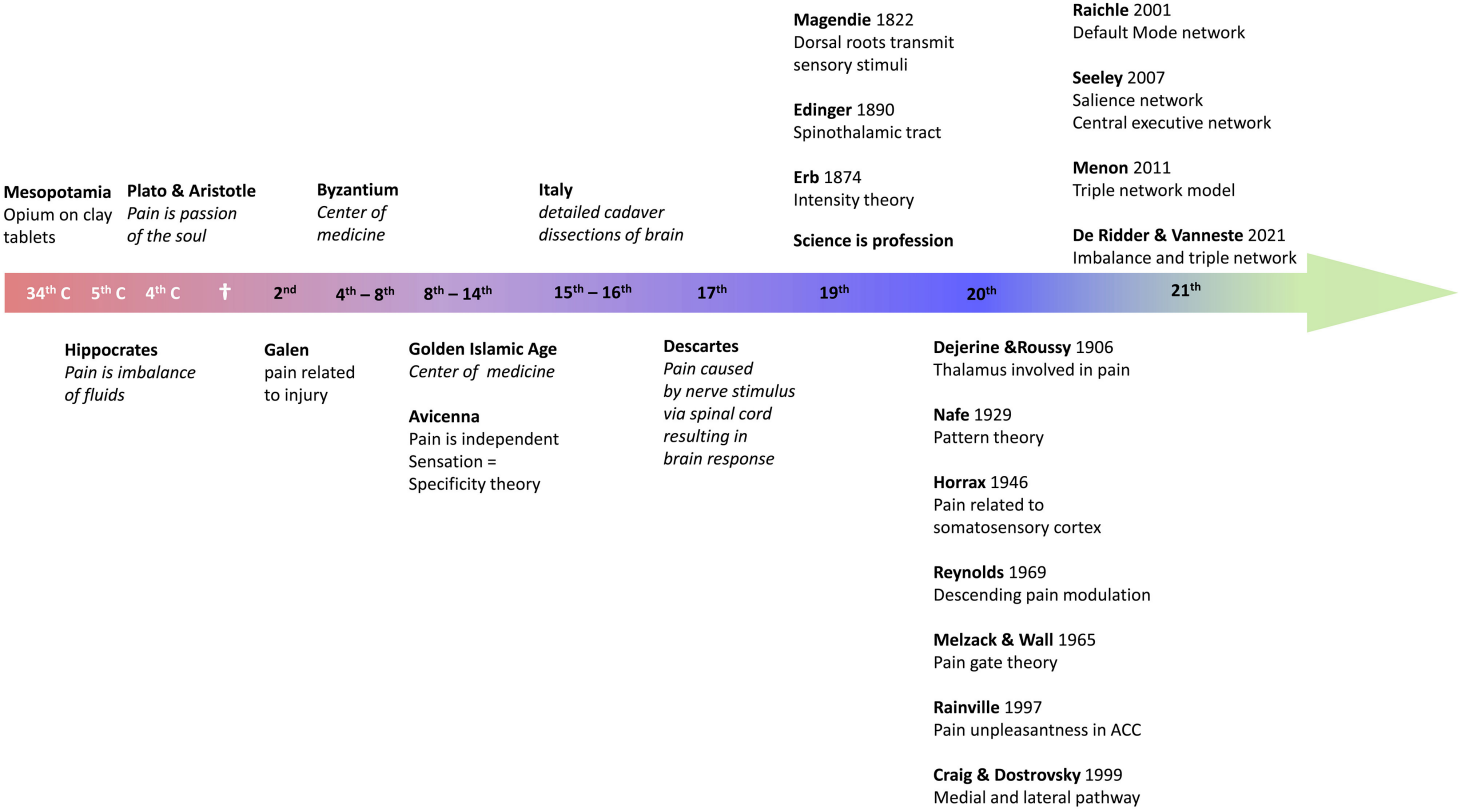
Overview of the history of pain research.

Pain has been treated by opioids ever since recorded history. Indeed, the Sumerian clay tablets written in cuneiform writing, dating back to 3,000 BC, in Mesopotamia, already mention opium (13). And Homer, in 800 BC, wrote in the Odyssey that Helen, the daughter of Zeus, gave Telemachus opioids to reduce the grief of Odysseus' absence (13). Plato and his pupil Aristotle considered pain to be an emotion, a passion of the soul (14). Hippocrates, the father of Western medicine, who lived in Greece from 460 to 377 BC, i.e., a contemporary of Plato and Pericles, was the first physician to believe that diseases were caused naturally, not because of superstition and gods (15). As such, he separated medicine from religion: disease was not a punishment inflicted by the gods but rather the result of environmental factors, diet, and living habits (15). His work is compiled in the *Corpus Hippocraticum*, which consists of 60 books. Hippocrates considered pain to be the result of an imbalance of the humors. Later, in the 2nd century AD Galen stated that pain was always the result of tissue injury (16). This view may result from the fact that he became the physician of the gladiators, as well as of the emperor Marcus Aurelius. Galen's voluminous work would dominate Western medicine for nearly 1,400 years. During the Islamic Golden Age from the 8th to the 14th century the center of medicine shifted to the Near East, North Africa and Spain. Avicenna (980–1,037) was the first to describe pain as a separate and specific sensation, giving rise to the specificity theory (14, 16). As an exponent of the scientific revolution in the 17th century Descartes provided a fully mechanistic explanation for pain, not much different from current insights in the pathophysiology of pain. In 1,664 he described an ascending pain pathway in which a peripheral stimulus was transmitted via peripheral nerve to the spinal cord and from there relayed to the pineal gland in the brain to reach consciousness (17). The 19th century saw the birth of science as a profession, and the term “scientist” was coined in 1,833 by the polymath William Whewell (1,794–1,866), replacing the old word “natural philosopher” (18). The first periodicals date from 1665, when the French *Journal des sçavans* and the English *Philosophical Transactions of the Royal Society* began to systematically publish research results. This professionalism created a wealth of new discoveries. In 1822 Magendie discovered that the dorsal nerve roots, and not the ventral roots, were transmitting sensory stimuli, and in 1890 Edinger described the spinothalamic tract (14). Erb in 1874 suggested that the specificity theory, dating back to Avicenna but further developed by Muller in 1840, might need to be replaced by the intensity theory, in which intense stimuli would activate sensory nerves that normally process other sensations to become painful (14). At the beginning of the 20th century Dejerine and Roussy proposed that the thalamus is involved in pain (14), and in 1946 Horrax stimulated the somatosensory cortex at high intensity, which generated pain (19). In 1969 Reynolds demonstrated that stimulation of the periaqueductal gray would modulate pain by activating a descending pathway (20), which triggered Melzack and Wall to propose the pain gate theory 1 year later (21). It took 30 more years before Rainville in 1997 demonstrated, using PET scans, that the affective component of pain was processed by the rostral anterior cingulate cortex (22), and quickly afterwards, in 1999, Craig and Dostrovsky unified the model of pain by detailing the medial and lateral pathways (23, 24). In 2021 De Ridder en Vanneste proposed that pain is an imbalance between the medial plus lateral pathway vs. the descending pathway (25, 26) (see Figure 1 for overview).

**Figure 2:**
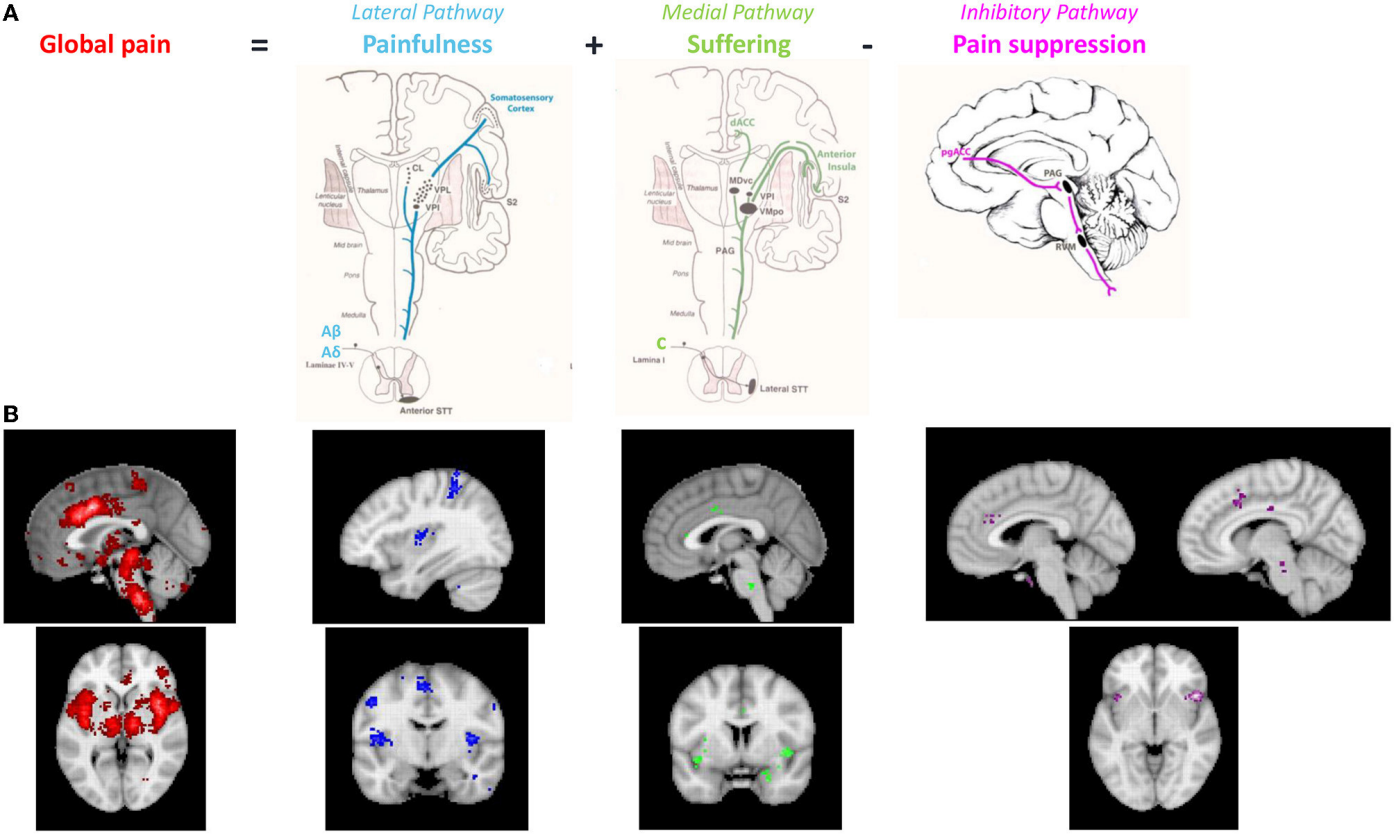
**(A)** The anatomical pathways associated with 3 different aspects of pain (painfulness, suffering and presence). **(B)** Neurosynth meta-analyses of functional imaging studies evaluating the different components of the pain signature in the brain. Meta-analysis of pain (*n* = 516 studies, red and blue), meta-analysis of suffering (*n* = 124, green) and meta-analysis of inhibition (*n* = 601, purple). Modified from reference De Ridder et al. (29).

**Figure 3:**
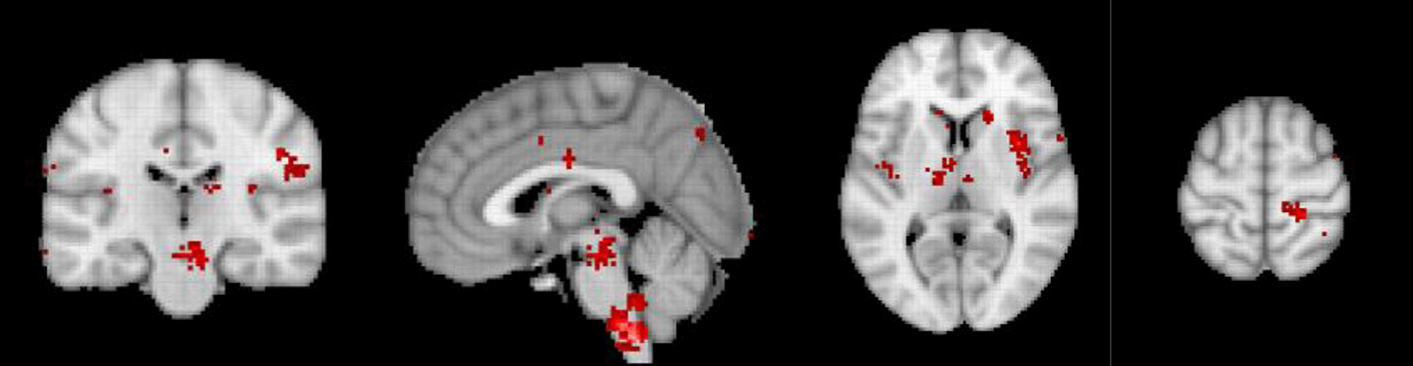
Neurosynth meta-analysis of chronic pain. The pre to subgenual anterior cingulate component is absent, suggesting that chronic pain is related to lost inhibition. Modified from reference De Ridder et al. (29).

Thus, it became evident that anatomically and symptomatically, chronic pathological pain can be dissociated into three separable but interacting pathways, comprising of a lateral “painfulness” pathway, a medial “suffering” pathway, and a descending pain inhibitory pathway (25, 27, 28) (Figures 2, 3). Whereas, the lateral somatosensory and medial salience pathway can explain the painfulness and emotional components of pain, no correlates have been proposed for the chronification and functional disability encountered in chronic pain. We propose extending the current pain networks to the triple network model to fill this gap.

**Figure 4:**
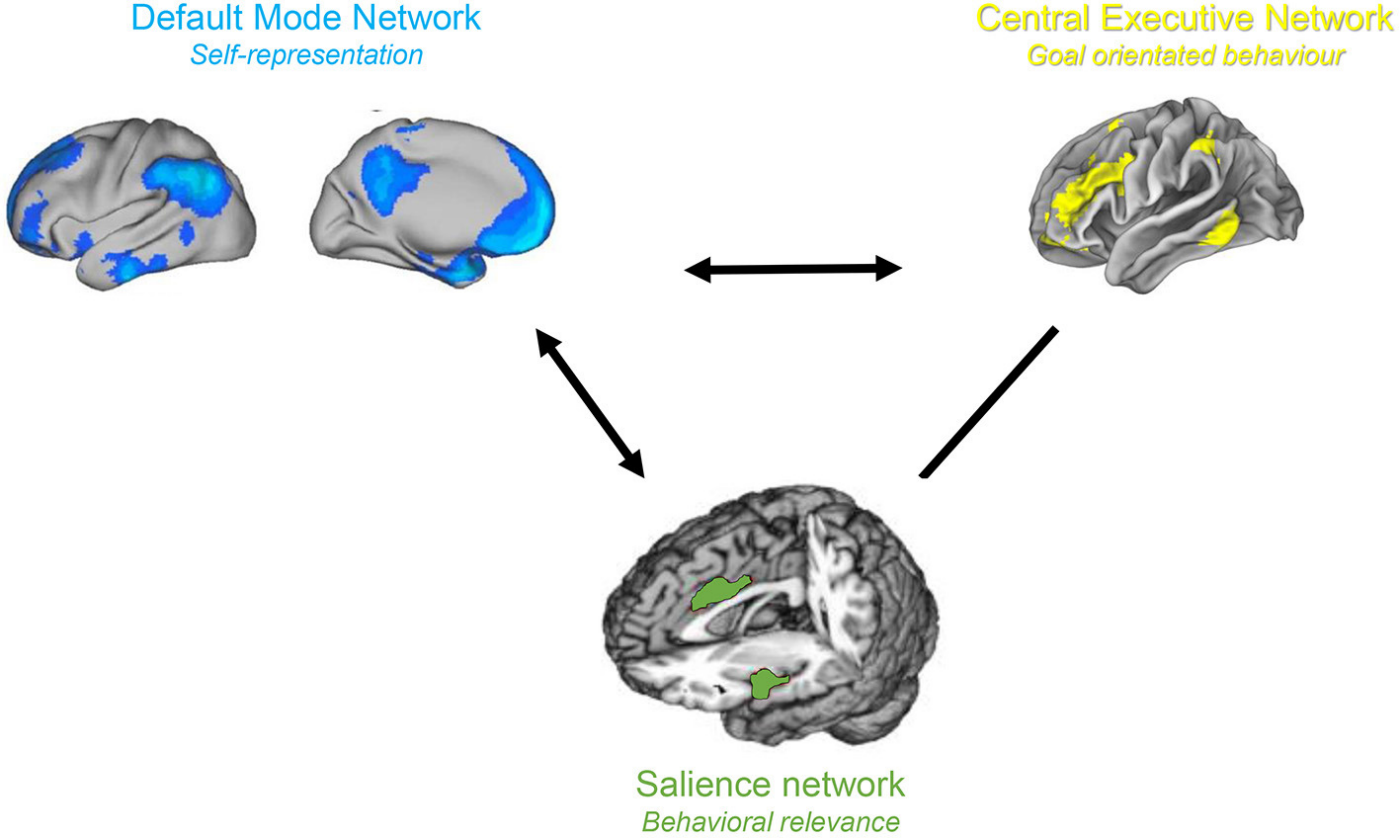
Triple Network model. The salience and central executive networks are correlated, and both are anti-correlated to the default mode network.

The triple network model is a network science based approach for studying cognitive and affective disorders (30). It claims that neuropsychiatric illnesses are caused by aberrant interactions within and between three cardinal brain networks. These three networks include the self-representational default mode network (31, 32), which was first described by Raichle in 2001 (33), the behavioral relevance encoding salience network (34) and the goal oriented frontoparietal central executive network (34, 35) both identified by Seeley in 2007 (34) (Figure 4). Normally, the central executive network and salience network demonstrate correlated activity, and both networks are anti-correlated to the default mode network (36). The salience network acts as a switch between the anticorrelated default mode network and the central executive network (37–39). This is in keeping with the proposed functions of the three networks. When the salience network identifies an external behaviourally relevant event it reduces the activity of the self-oriented and mind wandering default mode network and activates the external goal oriented central executive network to deal with the external salient stimuli. The functional connectivity within and between these three cardinal networks is abnormal in numerous brain disorder including as depression, anxiety, schizophrenia, and posttraumatic stress disorder (30). We propose that in chronic pain the three known pain pathways can be extended to the triple network model, which would explain chronification of pain as well as the commonly associated cognitive dysfunction.

### The Known Brain Anatomy of Pain

It is well-known that processing of pain related information involves four processes: transduction, transmission, modulation, and perception. A stimulus activates the different sensory receptors (transduction), which is transmitted to the sensory cortex (transmission), inducing sensation (40).

Information from the nociceptors in the skin is transmitted via the dorsal root ganglia to the dorsal horn. From there the information is relayed to the brain via the anterolateral system. The anterolateral system consists of 3 components, the spinothalamic tract (= spinal lemniscus), the spinoreticular tract and the spinotectal tract (41). The spinoreticular tract controls alertness and arousal in response to painful stimuli, and the spinotectal tract orients the eyes and head toward the nocuous stimuli. The spinothalamic tract is made up of 2 separable tracts, the anterior and lateral spinothalamic tract (41). The anterior spinothalamic tract is known as the conventional pain pathway, and because it relays to the lateral thalamic nuclei (VPL, VPI) (23, 24, 41, 42), it is also known as the lateral pain pathway (23, 24). It encodes for the discriminatory components of pain such as pain intensity, location, pain characteristics etcetera (29). Somewhat semantically confusing, the lateral spinothalamic tract, also known as the lamina I spinothalamic pathway (43), relays to the medial thalamic nuclei (mediodorsal and ventromedial posterior) (23, 24), and consequently it is also known as the medial pathway (23, 24). This pathway encodes the affective-motivational components of pain, in other words the suffering (29).

This sensation is further processed (modulation) by other brain networks such as the default mode, salience network and central executive network (= triple network) that brings the pain stimulus to consciousness (44–49), and creates an internal representation of the outer and inner world called a percept (40). Pain perception can thus be defined as “the act of interpreting and organizing a painful stimulus to produce a meaningful experience of the world and of oneself” (40).

When an individual reports that they are “in pain,” it actually involves three components, i.e., they have a certain amount of painfulness associated with a particular amount of suffering for specific amount of time. These three different components of the unified pain percept can be linked to the three different pain processing pathways (Figure 2).

The two ascending pain pathways consist of the anatomically and functionally separable medial and lateral pain pathways (28, 50–52). The medial pain pathway, consisting of the rostral to rdACC and anterior insular cortex, processes the affective motivational aspect of pain. The causality is demonstrated by the fact that cingulotomies interfere with negative affect and cognitive control (53). Correlational analysis furthermore relates the rdACC to unpleasantness (22, 28, 50, 52, 54). The lateral pain pathway, involving the somatosensory cortex (SSC) encodes the discriminatory/sensory components of the pain, such as painfulness, pain localization, and pain character (burning, aching, etc.) (28, 51, 55). A third pathway, namely the descending pain inhibitory pathway, balances the two ascending pain pathways (25, 26, 56, 57). The cortical areas involved in the descending pain inhibitory pathway include the rostral and pgACC, the periaqueductal gray, the parahippocampal area, hypothalamus, and rostral ventromedial brainstem (56–58). The descending pain inhibitory pathway controls context dependent pain perception (59), placebo analgesia (58, 60–62), and is deficient in pain syndromes characterized by generalized pain (63). Thus, the descending pain inhibitory pathway reflects the capacity of the brain to suppress acute or ongoing pain. A fMRI study of tonic spinal cord stimulation has shown that the amount of pain suppression is dependent on the amount of activation of the pgACC, part of the descending pain inhibitory system (64).

As described, the descending pain modulatory system, used to be thought of as the descending pain inhibitory system. Yet, it has become clear that this system actually can both inhibit or facilitate pain (65–68). Anatomically it starts from the DLPFC and runs via the pgACC to the reticular nucleus of the thalamus, periaqueductal gray, rostroventral medulla oblongata to the spinal cord, where it modulates the spinal gate (65, 69, 70). Its uses at least 4 different neurotransmitters: noradrenaline, serotonin, dopamine and opioids (56, 60, 65, 68). Pain (and suffering) can be regarded as the consequence of an imbalance between the two ascending and the descending pain inhibitory pathways (29). The exact mechanism of this balancing act is yet unraveled (65), but is likely under control of the reward system (25, 26, 29).

These anatomical pathways, if truly reflecting different clinical aspects of pain should be identifiable using functional imaging, both fMRI-based and EEG based. A neurosynth meta-analysis of pain based on 516 fMRI imaging studies (Figure 2B, red) shows a nice overlap between the functional BOLD activations and the three anatomical pathways, confirming the value of these anatomical dissociations (29). Furthermore, the medial and descending routes for distinct sensory modalities may be either non-specific or run in adjacent tracts. Indeed, a neurosynth meta-analysis of 124 functional imaging studies found that suffering, regardless of etiology, is linked to dACC and anterior insula activity, as well as the right supramarginal gyrus (Figure 2B, green). According to a neurosynth meta-analysis of 601 studies, inhibition in general, involves the salience network, and the pgACC to rACC, as well as the anterior component of the posterior cingulate cortex and right dorsal lateral prefrontal cortex, regardless of the stimulus that is being inhibited (Figure 2B, purple). This is a lateralized network, predominantly right-sided.

Using machine learning, a fMRI neural signature for acute pain has been developed with 94% accuracy (71). The neural signature includes the bilateral dorsal posterior insula, the secondary somatosensory cortex, the anterior insula, the ventrolateral and medial thalamus, the hypothalamus, and the dorsal anterior cingulate cortex (71), in other words components of the lateral and medial pathways. In contrast to neurosynth meta-analysis on (acute) pain, the same analysis of 92 studies on chronic pain does not show any activity in the descending pain inhibitory pathway. But the involvement of the lateral and medial pain pathway is still present. The comparisons of the neurosynth meta-analyses clearly suggests that chronic pain may be the result of a deficiency in activation of the pain suppression pathway, rather than the increased activation of the ascending pain pathways. This is in keeping with earlier reports that chronic pain is linked to lost thalamic inhibition (72) (Figure 3). The deficiency of the pgACC to rACC, in the development of chronic pain has been well-detailed in one form of generalized chronic pain: fibromyalgia (63, 73, 74). A similar purely data driven approach has also identified an EEG neural signature of chronic pain, with a similar accuracy of 93% (75), with almost similar areas.

In summary, clinical aspects of pain correlate with distinct anatomical pathways, which themselves corroborate with the functional imaging data. Furthermore, purely data driven machine learning confirms that pain can indeed be reduced to three hubs (pgACC, dACC, SSC) as expressions of the three pathways (descending, medial, lateral).

### Pain and Suffering Are Different

As mentioned, pain is defined as an unpleasant sensory and emotional experience associated with actual or potential tissue damage, or described in terms of such damage (1). Pain thus consists of painfulness with suffering. Suffering is an unpleasant or anguishing experience affecting an individual at a psychophysical and existential level, and is associated with negative cognitive, emotional, and autonomic responses to a (painful) stimulus. In other words, pain consists of physical pain associated with psychological pain, the first linked to the lateral pathway, the latter to the medial pathway.

The sensation of pain (fullness) can lead to suffering via the associated feeling of (emotional) unpleasantness and (cognitive) catastrophizing (76). Pain catastrophizing is characterized by (1) Magnification: the tendency to magnify the threat value of pain stimulus, (2) Helplessness: to feel helpless in the context of pain, and (3) Rumination: a relative inability to inhibit pain-related thoughts (77). In individuals with deficient cognitive coping strategies, pain catastrophizing could thus act as an amplifier on unpleasantness and pain intensity. Neuro-anatomically, pain catastrophizing is correlated with activity in the anterior superior part of the insula, i.e., the cognitive component of the insula (78, 79), and negatively correlated to the medial component of the default mode network (80).

**Figure 5:**
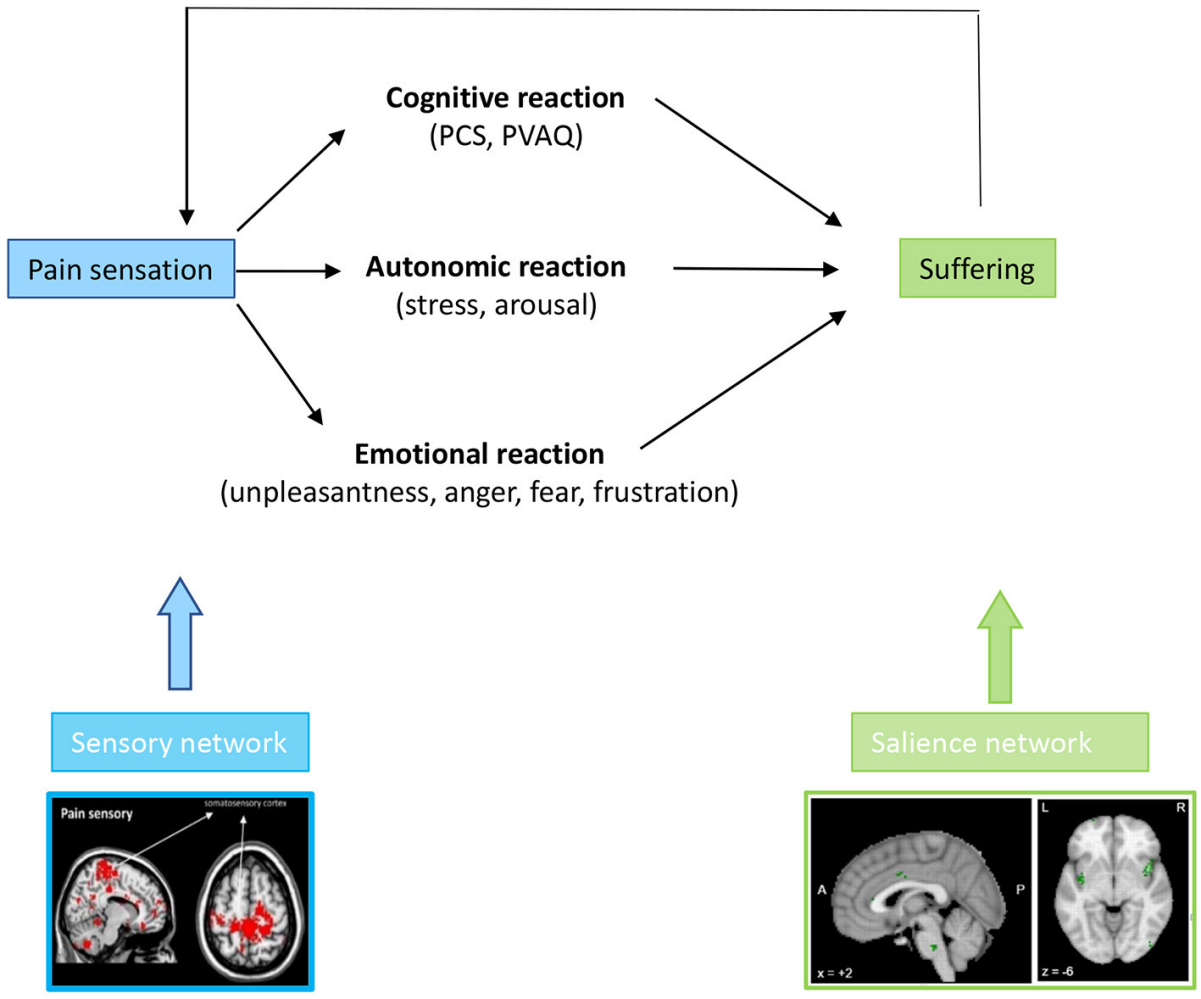
A chronic painful stimulus leads to a cognitive, emotional, and autonomic response, which phenomenologically expresses as catastrophizing, attention paid to the pain, unpleasantness, fear, anger or frustration with pain and arousal/distress. These cognitive, emotional, and autonomic symptoms are all phenomenological expressions of altered activity in the medial pathway.

The combination of the perceived unpleasantness and catastrophizing leads to suffering, which can express in different behaviors, including anger, fear, frustration, anxiety and depression (76, 81, 82) as well as functional disability (83) (Figure 5).

Pain is being used in research as a prototypical stressor (84). Physiological stress can be defined as an unpleasant sensory, emotional and subjective experience that is associated with potential damage of body tissue and bodily threat (85), especially when an environmental demand exceeds the natural regulatory capacity of an organism (86). The body responds to stress by an immediate adaptive short-lived response of the autonomic nervous system, followed by a slower extended activation of the hypothalamic-pituitary adrenal endocrine axis (87). Whereas, acute stress responses are adaptive and beneficial to survival by preparing a fight-or-flight response, chronic stress becomes maladaptive, leading to a host of problems including the metabolic syndrome, obesity, cardiovascular disease, mental health disorders, cancer, and increased susceptibility to infections (88). Acute stressors and threats activate the immune system in an adaptive way, homeostatically regulated by glucocorticoid negative feedback (89). Chronic stress results in a maladaptive immune response with an inflammatory predispositional vulnerability that has been implicated in stress-related psychopathology (89), including depression (88, 90), anxiety (90), and anger (91), in other words suffering.

The primary neural correlate of pain unpleasantness includes the rdACC, as demonstrated both in healthy volunteers (22, 92, 93) as well as chronic pain patients (93). The increased cortisol in stress results in unpleasantness by its modulation of the rdACC (93). In healthy participants, pain unpleasantness has also demonstrated positive correlations with anterior insula activity (94, 95). In contrast, in chronic pain patients, pain catastrophizing has demonstrated correlations with activity in the cognitive part of the insula, i.e., the dorsal anterior insula (80). A recent systematic review without meta-analysis in healthy controls as well as chronic pain patients, demonstrates that catastrophizing is not only associated with activity in the anterior insula, but also in the rdACC and somatosensory cortex (96). Thus, it is evident that chronic pain patients and healthy controls exhibit different neural correlates for both the pain unpleasantness and pain catastrophizing. The neural correlates of physiological stress, as identified by a meta-analysis also involve the rdACC and anterior insula (85).

The capability of independent modulation for both unpleasantness (suffering) and pain intensity (painfulness) (51), reveals that these two pathways are separable. Maneuvering the attentional state primarily alters the perceived pain intensity, without significantly altering the perceived pain unpleasantness (51). Comparatively, altering the mood state alters the perceived pain unpleasantness, without altering the pain intensity (51). This phenomena has been identified in the 1940s, as following frontal lobotomies performed for chronic pain it was observed that “The operation does not abolish physical pain but tends to change the menta1 attitude so that the patient does not suffer as he did before (97).” Similar observations were made following more focal lesions, as in cingulotomies for chronic pain (98), and are similar to changes noted with electrode implants in the rdACC (99, 100).

In summary, pain is made up of two aspects: the sensory painfulness aspect, encoded by the lateral pathway, and a suffering aspect, encoded by the medial system. Suffering has three components: cognitive, emotional and autonomic component, all of which are processed by the medial pathway. Because the medial and lateral pathways are distinct, one can experience pain (fullness) without suffering and suffering without pain (fullness).

### Pain, Suffering and the Salience Network

Observations made in wounded soldiers evacuated from the frontline in the Second World War clearly documented that there is no relationship between the extent of the injury and experienced pain (101). From an evolutionary point of view this makes sense. In the frontline of a battlefield surviving is more behaviorally relevant, i.e., more salient than pain perception. Indeed, pain can lead to immobilization, and thus prevent appropriate measures (fight or flight response) essential for staying alive. In Beecher's words, who evaluated the soldiers coming from the frontline: “The intensity of suffering is largely determined by what the pain means to the patient” (101), in other words “the intensity of the suffering is largely determined by the salience of the pain in this specific context.” As a result, the suffering, and in particular the unpleasantness is context dependent (59). The context of war, in which survival (natural selection) is more salient than suffering from pain also holds for the opposite. The context of sexual encounter, i.e., sexual selection, can also be more salient than pain. In sado-masochism, pain can be perceived as pleasant, the opposite of suffering. However, the discomfort is only considered pleasurable in the very specific erotic context (102). When a sadomasochist experiences tissue damage, e.g., hits his finger with a hammer, in a non-erotic situation, i., the pain will feel equally unpleasant and causes the same amount of suffering as for a non-sadomasochist (102). As a result, whether pain is linked with suffering or pleasure is determined by context (59, 102). In other words, the benefit of an equal pain stimulus is altered by context (59). Pleasant pain results from engagement of the descending pain inhibitory pathway and reward (accumbens and caudate) system, whereas unpleasant pain is associated with dACC and insula, in other words the medial “suffering” pain pathway (59), which overlaps with the salience network (29). Masochists also engage the descending and medial pain pathway (rdACC-insula) (102). The fact that the parahippocampal region, the primary hub in contextual processing, drives the transition from suffering to pleasure in the sadomasochistic context elucidates the contextual influence (103–107).

Context also modulates the placebo and nocebo effect, very common in pain therapies (108–111). The magnitude of placebo analgesia effects is highly variable and is determined by several contextual factors (112). Multiple meta-analyses have elucidated that the magnitude of the placebo pain suppressing effect is at least equally high as the intrinsic treatment effect (113–115) and can explain 75% (47–91%) of the therapeutic gain (114, 115) (= placebo + specific effect). The placebo effect depends on modulation of the three described pain pathways, as identified by multiple meta-analyses of the neural signature of placebo in pain studies (61, 116–118).

Physical pain has the ability to influence mood. A meta-analysis shows that acute physical pain might diminish negative affect (119), which may help to explain why self-harm can have a favorable effect in psychiatric diseases. Acute physical pain has the opposite effect in healthy controls in comparison to psychiatric patients: it worsens negative affect (120). Furthermore, when compared to healthy subjects, self-harmers have higher pain thresholds and pain tolerance, and report less pain intensity (121, 122).

### Pain Chronification, Energy Expenditure and the Default Mode Network

Network science is a branch of science that studies complex adaptive networks such as telecommunication, computer, economic, biological, social, cognitive, and semantic networks by breaking them down into nodes (or vertices) and connections (or edges). The use of network science to investigate the role of resting state network connections in brain disorders, such as chronic pain, is becoming more common (123–126), One of its findings is the involvement of the default mode network in chronic pain (127–129). The longer the pain exists, the stronger the connections between the primary somatosensory cortex and the default mode network (128). And this mechanism seems to be universal as it holds for CRPS, chronic back pain, and osteoarthtitic pain (128). The default mode network, which controls self-representational processing has been suggested to become pathologically coupled to pain provoking networks in chronic pain (130). The relevance of this finding is enormous, since it could provide a neurobiological explanation for why in chronic pain becomes embodied, that is, becomes an integral part of the self, making treatments more difficult. When the suffering is chronic, not only may the pain become a part one‘s identity, but fear can develop into worry/anxiety and the sadness into depression. In sustained anxiety the amygdala becomes coupled to the dorsomedial prefrontal cortex of the DMN (131, 132), and persisting inflexible sadness in depression is characterized by increased functional connectivity between the subgenual anterior cingulate cortex and the pgACC-rACC of the default mode network in contrast to controls (133).

This raises the question of why these somatosensory—default mode connections develop? The free energy principle of brain activity provides an evolutionary explanation (134, 135). In summary, it proposes that energy-intensive organs, such as the brain, will try to preserve energy in every way possible. The major component of the brain's parasympathetic nerve network overlaps with the default mode network (136), while the salience network and the sympathetic central control overlap in the brain (137).

**Figure 6:**
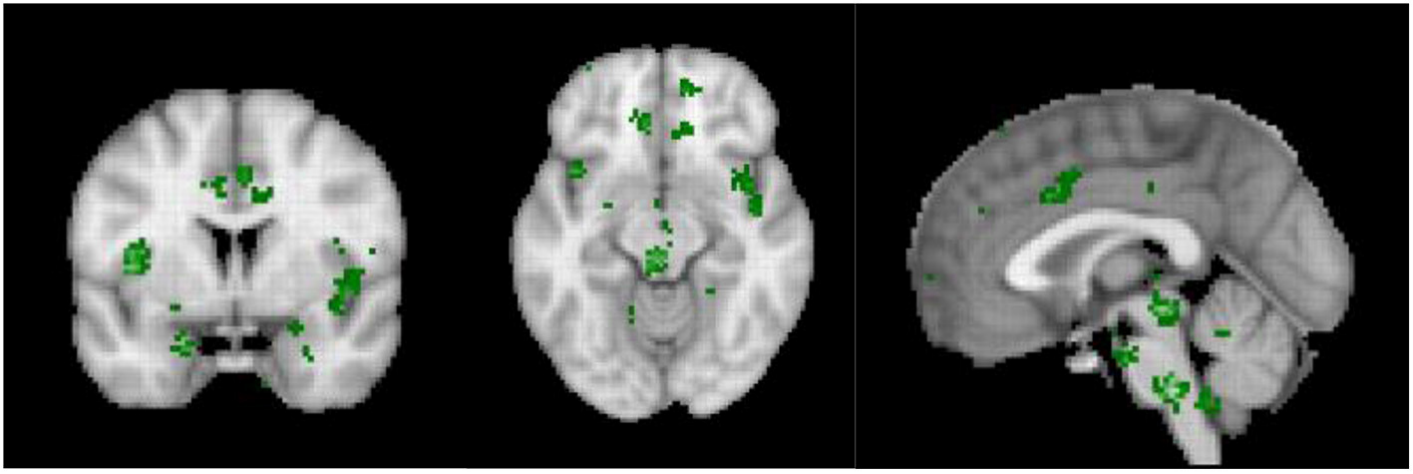
Neurosynth meta-analysis of the central autonomic network (*n* = 117 studies).

The sympathetic system increases energy consumption by 15–35% (138, 139). And indeed, in acute pain, the daily energy expenditure is increased by 60% (140), whereas in more chronic pain, the daily extra energy expenditure is only increased by 15% (141, 142). Similarly, fear increases the energy expenditure by 22% (143), whereas chronic anxiety only increases energy expenditure by 6% (144). Energy expenditure could be reduce by rewiring the pain pathways to connect to the default mode network, which overlaps with the parasympathetic central network and disconnect from the energy consuming sympathetic nervous system. The differences in the meta-analyses between acute and chronic pain, as indicated above provide indirect evidence. It can be noted that the central hub of the sympathetic nervous system, i.e., the dACC (145) is deactivated in chronic pain in contrast to acute pain, thereby saving energy. Yet, interestingly, even though the central sympathetic network (Figure 6) may be disconnected from the pain, the arousal state, i.e., the stress remains, suggesting that allostatic mechanisms, characterized by reference resetting, may be involved (29). As such, even a low electrophysiological arousal state may have stress as an emergent property.

### Pain, Cognitive Dysfunction, and the Central Executive Network

**Figure 7:**
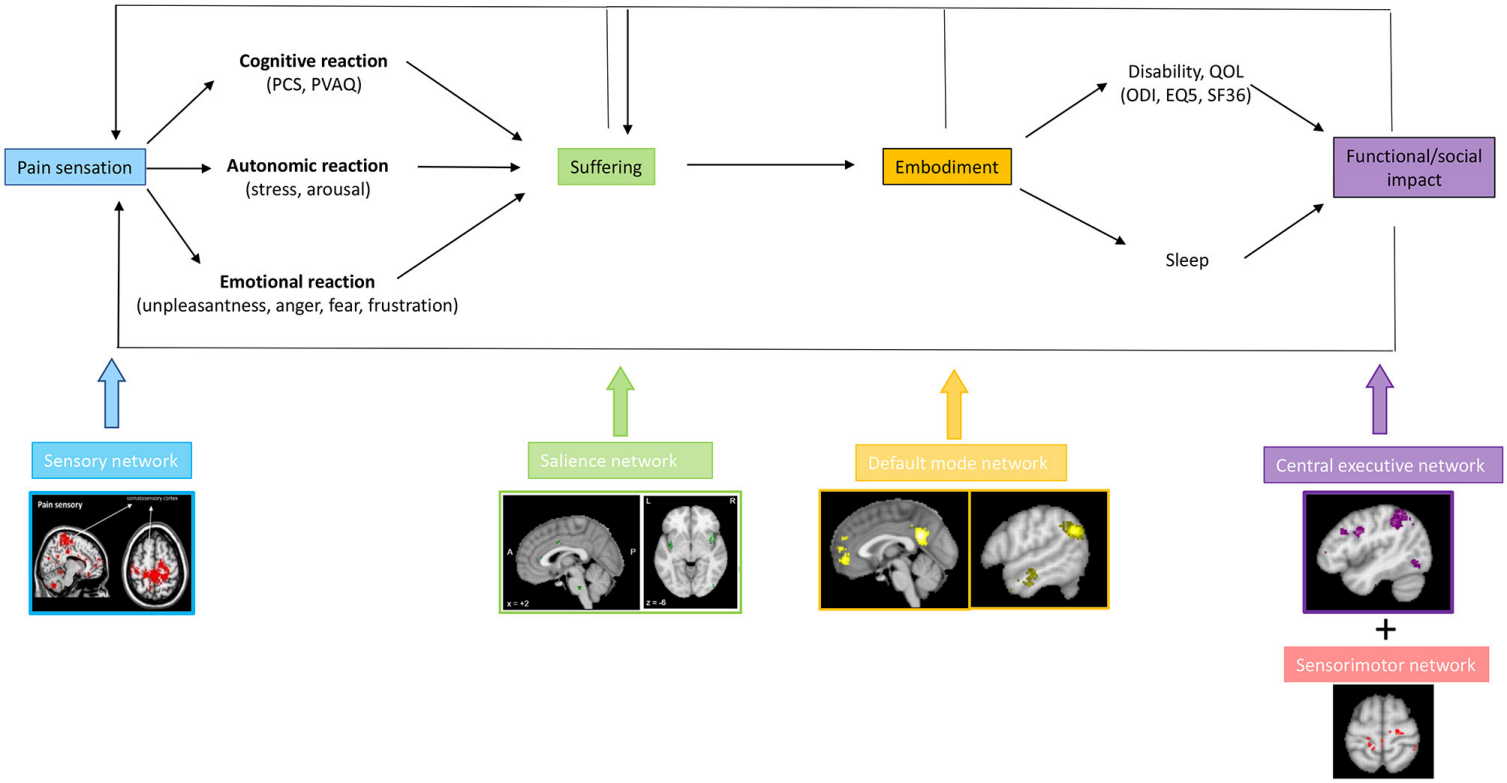
The presence of a painful stimulus in the lateral somatosensory pathway, can lead to a cognitive, emotional, and autonomic response, encoded by the medial salience pathway expressed by suffering. When the pain and suffering become chronic, they become embodied, i.e., part of the self, mediated via connectivity of the somatosensory cortex to the default mode network. The embodied pain and suffering can subsequently lead to physical and cognitive disability, possibly mediated via dysfunctional connectivity with the motor and the central executive network, respectively.

Chronic pain can lead to suffering, and this consequently can lead to a decrease in quality of life and the development of pain associated disability, both physical and cognitive disability (76, 81, 146). Based on network science principles it can be envisaged that each aspect of pain is the result of connectivity changes between the lateral pathway, i.e., somatosensory network, and another resting state network, such as the salience network (suffering), the default mode network (embodiment) and central executive network (cognitive disability) and motor network (physical disability) (Figure 7). Patients with failed back surgery syndrome (147), migraine (148) and low back pain (130) are characterized by changes in the sensorimotor, salience, default mode and central executive networks. However, in these studies, no correlation analyses were performed to link these network changes to clinical syptoms or comorbidities, limiting the interpretation of what these changes mean. The default mode network is anti-correlated with the salience network, which overlaps with the medial pathway and the stress network in healthy subjects (36). In chronic pain this anti-correlation is lost (149). Indeed, chronic low back pain is characterized by hyperconnectivity of the primary somatosensory cortex to the default mode and executive control network (130). These somatotopic linkages between the somatosensory “painfulness” network and the “suffering” salience network, as well as the default mode, are limited to the homuncular cortical representation of the painful body area (127). This makes logical sense because only the pain from the damaged area becomes part of the self, allowing the sympathetic system to be activated for pain from other parts of the body. Pain is associated with increased connection between the default mode network and the anterior insula of the salience network, which are generally anticorrelated. The concept that chronic pain is linked to the progressive engagement of multiple resting state networks necessitates research that links specific clinical characteristics of pain to activity and connectivity metrics. Furthermore, if this hypothesis is right, large-scale research involving patients with and without suffering, with and without embodiment of pain and suffering, and with and without handicap may not be appropriate. From a theoretical point of view, it can be hypothesized that painfulness below a certain threshold, e.g., a NRS of 4–5 only results in painfulness, without suffering. This would show a limited network activation of functional imaging. When the pain level rises to 6/10, functional connections between the lateral and medial circuits may begin to cause suffering. Increased connectivity to the central executive network may result in functional incapacity once the pain reaches a higher value, such as 7/10. If this concept is right, more customized therapies for chronic pain could be developed.

The clinical implications of this approach are clear. Whereas, the therapeutic implications of targeting the medial, lateral and descending pathways have been described (29), extending the model to the triple network are evident, especially by the use of neuromodulatory techniques such as brain stimulation and neurofeedback. Up to recently, traditional neurostimulation was phrenologically targeting one area at a time, and due to technological limitations, this is still the case for repetitive transcranial magnetic stimulation. Yet, transcranial electrical stimulation can theoretically target multiple areas at the same time, permitting multifocal or network neuromodulation (150). The theoretical underpinnings of how to modulate these networks have been described for future neuromodulatory approaches (151). As such, modulating the triple network is possible, but has not yet been attempted. These neuromodulatory techniques, if shown to benefit pain suppression, may be one of the factors that can help reduce the opioid crisis (152).

## Conclusion

Pain is handled by three separate yet interconnected networks, each of which encodes a different aspect of pain. The lateral pathway, which has the somatosensory cortex as its main hub, is primarily responsible for painfulness processing. The suffering component is related to medial pathway involvement, with the rdACC and insula as primary hubs, and the descending pain inhibitory pathway is possibly related to the proportion of time that pain is present. Pain (fullness) can exist without suffering, and suffering can exist without pain (fullness). Pain becomes part of the self-perception, part of who you are, when pain sensation pathways become correlated to the default mode rather than anticorrelated. By interfering with the goal-oriented central executive network, pain can lead to functional impairment. As a result, the three previously established pain pathways must be expanded to include the triple network in order to fully explain the full clinical picture of chronic pain.

## Author Contributions

DR created the concept and wrote the first draft. SV, MS and DA revised, improved, and rewrote the first draft. All authors contributed to the article and approved the submitted version.

## Conflict of Interest

The authors declare that the research was conducted in the absence of any commercial or financial relationships that could be construed as a potential conflict of interest.

## Publisher's Note

All claims expressed in this article are solely those of the authors and do not necessarily represent those of their affiliated organizations, or those of the publisher, the editors and the reviewers. Any product that may be evaluated in this article, or claim that may be made by its manufacturer, is not guaranteed or endorsed by the publisher.

## Abbreviations

BOLD: blood oxygen level dependent
dACC: dorsal anterior cingulate cortex
DMN: default mode network
EEG: electroencephalography
fMRI: functional Magnetic Resonance Imaging
NRS: numeric rating scale
pgACC: pregenual anterior cingulate cortex
rACC: rostral anterior cingulate cortex
rdACC: rostral to dorsal anterior cingulate cortex
SSC: somatosensory cortex.
